# Association between apolipoprotein B XbaI polymorphisms and coronary heart disease: A meta-analysis

**DOI:** 10.1186/s12872-020-01545-7

**Published:** 2020-06-03

**Authors:** Ya Yun Feng, Lu Yang Chen, Yang Liu, Meng Luo, Tian Tian Yang, Yu Hao Hu, Jing Chang, Min Mao

**Affiliations:** Department of Cardiology, The First Affiliated Hospital of Chongqing Medical University, Chongqing, China

**Keywords:** Apolipoprotein B, Polymorphism, Coronary heart disease, Meta-analysis

## Abstract

**Background:**

To evaluate the association between apolipoprotein B gene polymorphism and coronary heart disease in some populations at home and abroad by means of meta-analysis.

**Methods:**

Using the strict exclusion criteria for primary screening of the literature and applying the Hardy-Weinberg equilibrium to test the genetic balance of the selected literature. The corresponding models were selected according to the results of the heterogeneity test. The Begg’s test and Egger’s test were used to evaluate publication bias, and meta-analysis was performed using Stata 12.0.

**Results:**

The study included twelve articles. In the literature, a total of 1596 patients with coronary heart disease and 1431 controls.Meta-analysis results showed no statistical value in the following three genetic models: allelic comparison (a vs A,*P* = 0.811,OR = 0.95, 95%CI = 0.62–1.46), recessive genetic models (aa vs Aa/AA, *P* = 0.86,OR = 0.94, 95%CI = 0.45–1.96), or dominant genetic models (aa/Aa vs AA, *P* = 0.73,OR = 0.92, 95%CI = 0.58–1.47). Subgroup analysis based on ethnicity showed allelic comparison (a vs A,*P* = 0.464,OR = 1.32, 95%CI = 0.63–2.78), recessive genetic models (aa vs Aa/AA, *P* = 0.422,OR = 1.52, 95%CI = 0.55–4.21), and dominant genetic models (aa/Aa vs AA, *P* = 0.551,OR = 1.26, 95%CI = 0.58–2.73) in Asians, allelic comparison (a vs A,*P* = 0.410,OR = 0.79, 95%CI = 0.45–1.39), recessive genetic models (aa vs Aa/AA, *P* = 0.041,OR = 0.75,95%CI = 0.57–0.99),dominant genetic models (aa/Aa vs AA, *P* = 0.385,OR = 0.75, 95%CI = 0.40–1.43) in Caucasian;

**Conclusion:**

The ApoB(apolipoprotein B) XbaI locus is not a risk factor when it comes to the development of coronary heart disease in the domestic and international populations included in this paper. In Caucasians, people carrying the aa genotype may be less susceptible to CHD (coronary heart disease). The results of recessive genetic models have to take the effect of heterogeneity and sample sizes into account. Further research may require a larger and more rigorous research design.

## Background

As countries grow richer, cases of coronary heart disease(CHD) also rise rapidly, and coronary heart disease patients are becoming younger and younger. CHD is a major cause of both death and disability in developed countries, responsible for about one-third or more of all deaths in individuals over the age of 35 [1–3]. The 2018 Heart Disease and Stroke Statistics update of the American Heart Association reported that 16.5 million people over the age of 20 years old in the United States have suffered some form of CHD [3]. The etiology and pathogenesis of CHD have not yet been clearly clarified. At present, it is generally believed that the occurrence of CHD is the result of a series of risk factors and multiple susceptible genes. However, these environmental risk factors do not fully explain the incidence of CHD. Numerous epidemiological studies show that genetic factors are still the main cause of coronary heart disease. As we know, hyperlipidemia is one of the independent risk factors for CHD. Apolipoprotein B (ApoB) is the main component of low-density lipoprotein (LDL) in plasma, and ApoB polymorphisms constitute good predictors of CHD by regulating the metabolism and utilization of LDL [4].

ApoB XbaI (rs693) is a kind of single-nucleotide polymorphism (SNP) resulting in the change of amino acids, which is located in exon 26 of the ApoB gene. Many studies have been done on the correlation between ApoB XbaI gene polymorphism and CHD in different countries, however, their conclusions go different ways. Different studies at home and abroad have disputed the relationship between ApoB XbaI gene polymorphism and coronary heart disease, mainly because of the small sample size and genetic differences among different ethnic groups. Due to the limitations of a single study regarding research methods, number of studies, ethnicity of the study population and other aspects, it is important to summarize the results of these individual studies to be able to make reliable conclusions concerning XbaI gene polymorphism. The purpose of this meta-analysis is to integrate the results of these case-control studies to comprehensively assess the association between ApoB XbaI gene polymorphisms and CHD.

## Methods

### Literature search and databases

We have conducted a comprehensive search through the computer system in the following databases up until April 2019:MEDLINE database(http://www.ncbi.nlm.nih.gov/pubmed), Google Scholar(http://www.scholar.google.com),Web of Science (http://www.isiknowledge.com), Cochrane Library (http://www.thecochranelibrary.com),Chinese VIP database (http://www.cqvip.com),Chinese Wanfang database (http://www.wanfangdata.com.cn),China National Knowledge Infrastructure (CNKI) database (http://www.cnki.net). The search was limited to Chinese and English articles,for all publications about the association between ApoB XbaI gene polymorphisms and CHD, while excepting reviews and critical articles. The following key words were used while searching:apolipoproteins B/ApoB, ApoB XbaI polymorphisms, and coronary heart disease/coronary &disease/coronary disease &heart/ coronary atherosclerotic heart disease/CHD/CAD.

### Selection criteria

Articles were identified based on the inclusion criteria as follows: (1) The original study type was a well-designed case-control study with strict controls;(2) The studies should have exact genotypic and allelic data of ApoB XbaI;(3)Cases in the CHD group were diagnosed and cases in control group were excluded by coronary angiography, based on the World Health Organization (WHO) 1979 CHD Criteria;(4)The studies had comprehensive statistical indicators, such as odds ratio, confidence interval, etc. The genotypes of the case group and the control group in each study were in line with the Hardy-Weinberg equilibrium (HWE) to verify the representativeness of the study population, and if HWE-p was bigger than 0.05,which meant the research group met HWE, then it was included. The exclusion criteria of these articles were as followed:(1)The article was not a case-control study,but a lecture, review, correspondence, editorial letter,etc.;(2) Unable to extract exact genotype data from the article;(3) If the HWE-p was smaller than 0.05,which meant that the research group didn’t meet the Hardy-Weinberg equilibrium, it was excluded.

### Quality assessment

The quality of these case-control studies was assessed by a star rating system, Newcastle-Ottawa scale (NOS), and evaluation of the NOS score of the studies was averaged by two independent reviewers. Full score is 9 stars,while a score range from 0 to 4 stars is considered poor quality [5, 6],and a score range from 5 to 9 stars is considered high quality [7, 8]. The score of the studies was evaluated by the following items:(1)The definition of cases;(2)Representativeness of cases;(3)Selection of controls;(4) Definition of controls;(5)Comparability of cases and controls on the basis of design or analysis;(6)Exposure assessment, including ascertainment of exposure and non-response rate.

### Data extraction

Data of selected studies was extracted by two independent reviewers in a standard data collection form, which included the following items:(1)the basic information of the included studies,such as the first author’s name,the publication year, etc.;(2)the region of the study population;(3) genotype data and allele frequency of ApoB XbaI for both CHD cases and control cases;(4) HWE-p in healthy control groups. In case of disagreement, we settle it through discussion or refer to a third party for help.

### Statistical analysis

The statistical analysis of this meta was mainly performed using Stata 12.0, including the analysis and collation of data, and the obtainment of the frequency and OR value of the allele and its 95% confidence interval, so as to carry out the correlation between ApoB Xbal gene polymorphism and CHD. The Hardy–Weinberg equilibrium (HWE) was evaluated for each study by conducting Chi-square tests in control groups, and *P* < 0.05 was considered a significant departure from HWE. At the same time, I^2^ statistics were used to test the heterogeneity of each piece of literature [9]. If there is no obvious heterogeneity between the studies, the fixed-effects model is used for analysis. Otherwise, the random effects model is used to summarize the data [10].The potential publication bias is done using a funnel plot. The Egger test is used to quantify the publication bias [11] of the funnel plot. Finally, the stability of the experiment is evaluated by excluding a document to calculate whether the result changes.

## Results

### Characteristics of eligible studies

**Fig. 1:**
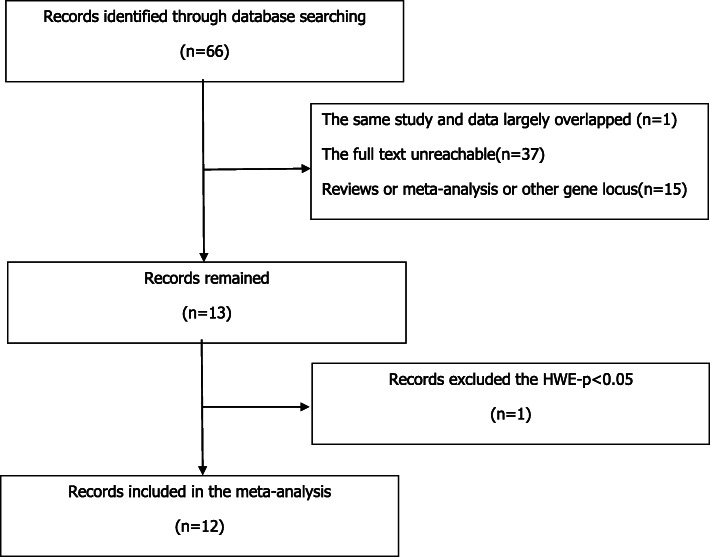
The detailed screening process

A total of 66 abstracts both in English and Chinese from the last 20 years on the association between apolipoprotein B Xbal polymorphisms and CHD susceptibility were identified, including 9 in Chinese and 57 in English. Whenever two studies largely overlapped [12, 13], only the most recent one [12] is included. When retrieving further, the full texts were available only regarding 28 of the remaining 65 abstracts. These full texts were read carefully, and 15 of them were ineligible to be excluded according to the inclusion criteria of this meta, such as case-control study, with genotypic data, diagnosis of CAD by angiography and with statistical indicators, etc. Literature in the form of meta-analysis and review was excluded, and literature with the data of gene frequency but not the exact genotype was also excluded. The 13 remaining studies were subjected to HWE test and one study [14] was excluded as the results showed significant deviations from HWE in the healthy control group (*p* < 0.05). Hence, 12 studies remained [12.15–25] and were used in this meta-analysis with a total of 1596 CHD patients and 1431 healthy control subjects. The detailed screening process was shown in Fig. 1 and the characteristics of the included studies were summarized in Table 1.Age, gender, hypertension, diabetes, body mass,etc.,were not evenly distributed in these 12 studies, so no information on these covariates were reported in Table 1. The quality of these studies was rated according to the NOS calculation system, and it was shown that the scores of these 12 studies were moderate or higher.

**Table 1:** Characteristics of the eligible studies in this meta-analysis

Author	Year	Region	Case AA	Case Aa	Case aa	Control AA	Control Aa	Control aa	HWE-p
Abd El-Aziz TA [15]	2016	Egyptian	92	34	6	63	63	9	0.1967
LI.S.H [17]	2013	Chinese	18	2	1	43	2	0	0.8788
Huang G [24]	2012	Chinese	165	35	5	190	42	4	0.3529
MA.J [16]	2011	Chinese	80	9	0	55	22	1	0.4617
Anghebem [22]	2009	Brazilian	93	113	45	46	76	26	0.5747
BELGIN [18]	2005	Turkish	47	85	18	46	46	8	0.4516
Marcos [20]	2001	Brazilian	17	37	13	24	35	8	0.3769
Luis A [21].	2000	Brazilian	42	48	10	12	34	54	0.0812
VADIM.A [23]	1998	Russian	31	55	32	25	49	18	0.4933
Seung [25]	1997	Korean	209	25	1	195	21	0	0.4527
Ju-Pin [19]	1995	Chinese	144	4	0	151	2	0	0.9351
YE.P [12]	1994	Chinese	64	16	0	59	1	0	0.9481

### Heterogeneity assessment

**Fig. 2:**
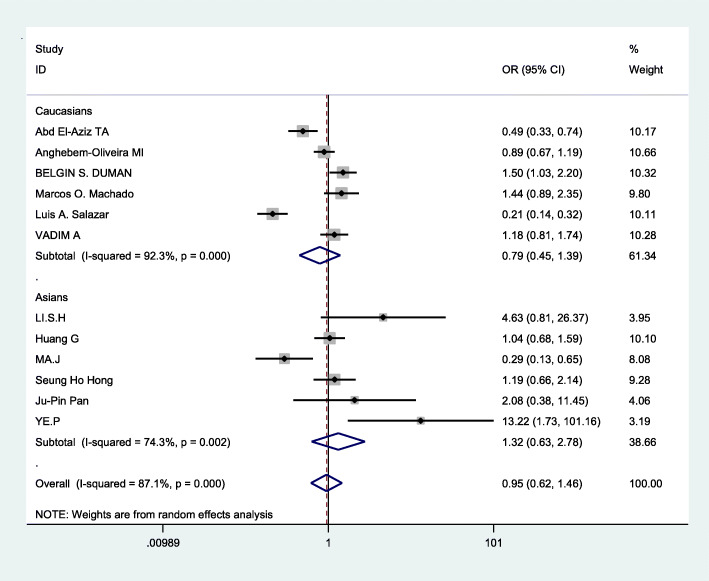
Forest plot of the association between CHD and ApoB XbaI subgroup analysis(recessive genetic models:aa vs Aa/AA)

The Q test has certain defects, which are mainly affected by the number of studies. The less number of studies, the lower test efficiency,and then I^2^ statistic was used to assess the heterogeneity respectively by using STATA12.0. In the three genetic models (allelic comparison, recessive genetic models, and dominant genetic models), significant heterogeneity was found: allelic comparison(I^2^ = 87.1%,*p* < 0.0001), recessive genetic models(I^2^ = 79.2%,*p* < 0.0001),dominant genetic models (I^2^ = 81.1%,*p* < 0.0001)(Table 2).Therefore, the random effects model was applied to merge data.Next, we used subgroup analysis according to ethnicity to analyze the source of heterogeneity in the three models. From Fig. 2 and the Table 3, We drew the conclusion that in recessive genetic models, ethnicity may be a part source of heterogeneity, but in other genetic models there was no significant evidence that ethnicity was the source of heterogeneity.

**Table 2:** Heterogeneity assessment by I^2^ statistics in the three genetic models

Genetic models	I^2^	p	Effect model
Allelic models(a vs A)	87.1%	< 0.0001	Random effects model
Recessive gene models(aa vs Aa/aa)	79.2%	< 0.0001	Random effects model
Dominant gene models(aa/Aa vs AA)	81.1%	< 0.0001	Random effects model

**Table 3:** Summary of polled ORs and I^2^ in the meta-analysis

	I^2^	OR(95%CI)	P	I^2^	OR(95%CI)	P	I^2^	OR(95%CI)	P
Genetic models	a vs A			aa vs AA/Aa			aa/Aa vs AA		
Over all	87.1%	0.95(0.62–1.46)	0.811	79.2%	0.94(0.45–1.96)	0.860	81.1%	0.92(0.58–1.47)	0.73
Asians	92.3%	1.32(0.63–2.78)	0.464	0.0%	1.52(0.55–4.21)	0.422	73.4%	1.26(0.58–2.73)	0.551
Caucasians	74.3%	0.79(0.45–1.39)	0.410	87.5%	0.75(0.57–0.99)	0.041	86.9%	0.75(0.40–1.43)	0.385

### Data merging

**Fig. 3:**
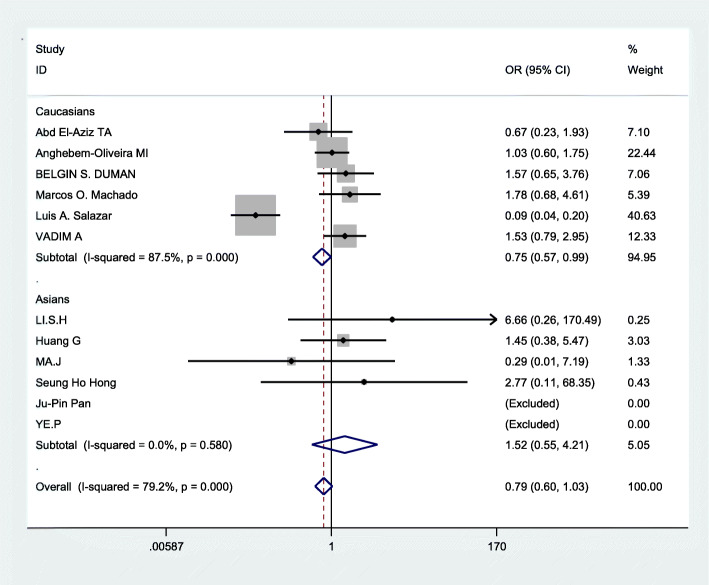
Forest plot of the association between CHD and XbaI for a/A allele. Odds ratios (OR) and 95% confidence intervals are shown. In the last line the over I2 statistic and p-value for heterogeneity are reported, which is 87.1% by I^2^ test(*P* = 0.000) (CHD: coronary heart disease; CI: confidence interval; OR: odds ratio)

**Fig. 4:**
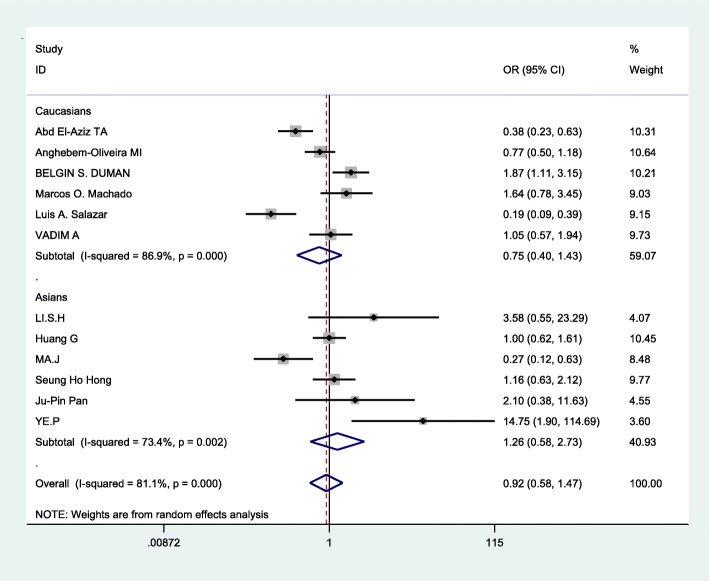
Forest plot of the association between CHD and XbaI for recessive genetic models:aa vs Aa/AA

**Fig. 5:**
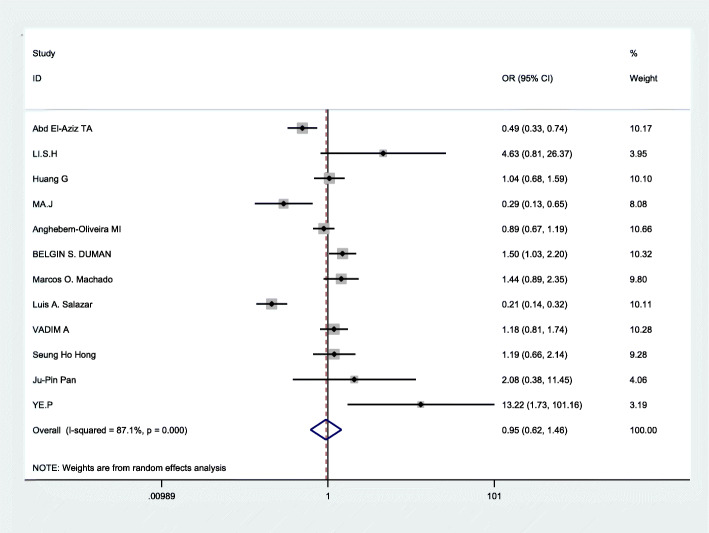
Forest plot of the association between CHD and XbaI for dominant genetic models:aa/Aa vs AA

**Fig. 6:**
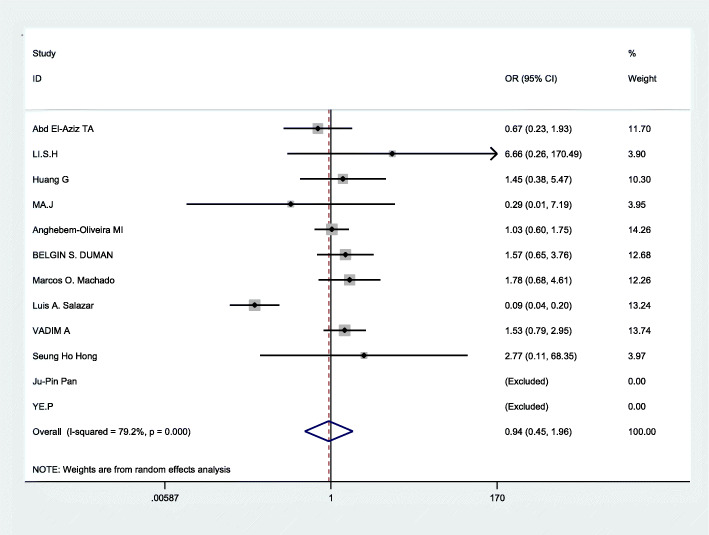
Forest plot of the association between CHD and ApoB XbaI subgroup analysis(allelic gene models:a vs A)

**Fig. 7:**
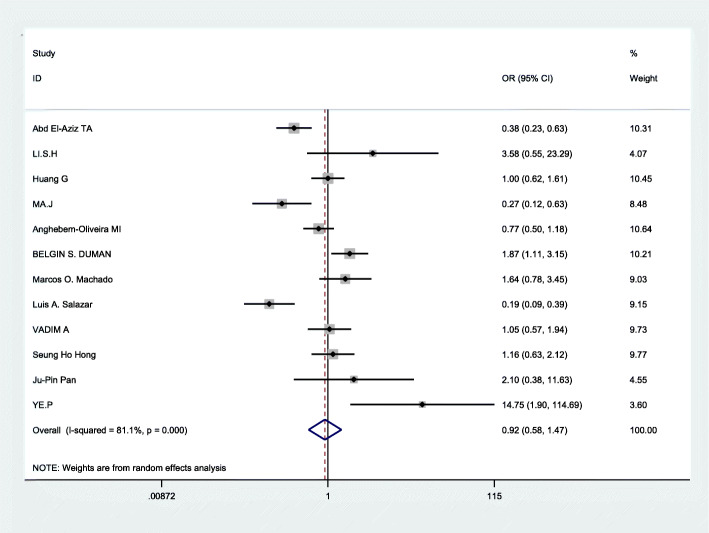
Forest plot of the association between CHD and ApoB XbaI subgroup analysis(dominant genetic models:aa/Aa vs AA)

Based on the statistically significant heterogeneity which was found for the analysis of allelic comparison, recessive genetic models, and dominant genetic models in this meta, the random effects model was employed to merge data. The combined OR values were calculated and the forest plot of the association between ApoB XbaI gene polymorphisms and CHD was made by D-L method, shown in Figs. 3, 4, 5 and Table 3. We know that there was no statistical difference in allelic comparison (a vs A,*P* = 0.811,OR = 0.95, 95%CI = 0.62–1.46), recessive genetic models (aa vs Aa/AA, *P* = 0.86,OR = 0.94, 95%CI = 0.45–1.96), or dominant genetic models (aa/Aa vs AA, *P* = 0.73,OR = 0.92, 95%CI = 0.58–1.47). Next, subgroup analysis was conducted (Figs. 6, 2, 7). In Asians, obvious heterogeneity was found in both allelic comparison (I^2^ = 74.3%, *p* < 0.0001) and dominant genetic models (I^2^ = 73.4%, *p* = 0.002), so therefore random effects model was used. While fixed effects model was used in recessive genetic models(I^2^ = 0.0%, *p* = 0.58).No statistical association was found to be significant in any genetic models: allelic comparison (a vs A,*P* = 0.464,OR = 1.32, 95%CI = 0.63–2.78), recessive genetic models (aa vs Aa/AA, *P* = 0.422,OR = 1.52, 95%CI = 0.55–4.21), or dominant genetic models (aa/Aa vs AA, *P* = 0.551,OR = 1.26, 95%CI = 0.58–2.73).In Caucasians,random effects model was conducted because of significant heterogeneity in any genetic models. Statistical significance was found in recessive genetic models (aa vs Aa/AA, *P* = 0.041,OR = 0.75,95%CI = 0.57–0.99),which meant that ApoB XbaI gene polymorphism had a certain correlation with CHD. However, no statistically significant difference was found in the other two genetic models(allelic comparison (a vs A,*P* = 0.410,OR = 0.79, 95%CI = 0.45–1.39), dominant genetic models (aa/Aa vs AA, *P* = 0.385,OR = 0.75, 95%CI = 0.40–1.43)). The above data showed that among Caucasians, those carrying the aa genotype may be less susceptible to CHD.

### Publication bias

**Fig. 8:**
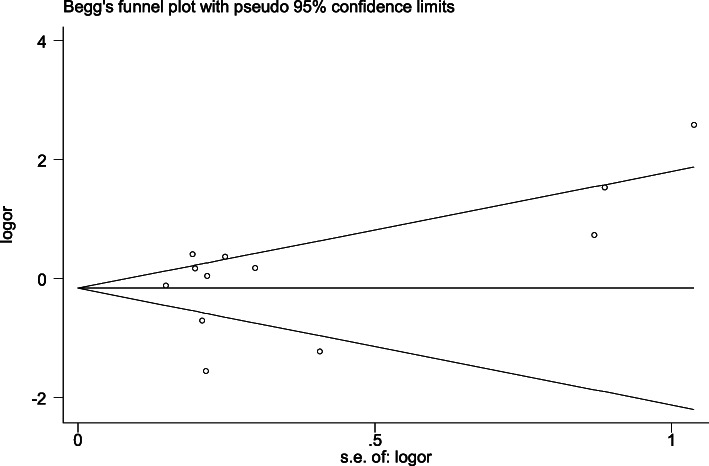
Funnel plot of the association between log odds ratio (logOR) and the standard error (SE) for the relationship between CHD and ApoB Xbal gene for A/a allele genotype

**Fig. 9:**
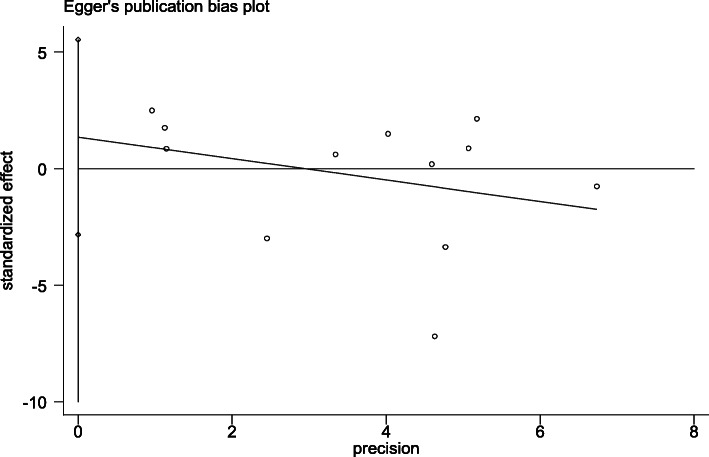
Funnel plot of the association between log odds ratio (logOR) and the standard error (SE) for the relationship between CHD and ApoB Xbal gene for A/a allele genotype

The Begg’s funnel plot test and Egger’s linear regression test were used to evaluate the publication bias of the above models(Table 3,Table 4). There was no publication bias regarding the association between ApoB XbaI polymorphism and CHD. In allelic comparison, the result of the Begg’s test showed that the *p*-value was 0.451,while the result of Eggers linear regression test showed that the p-value was 0.49 (Fig. 8). In another words, it meant that the results from the two different methods were consistent with *P* > 0.05,indicating no significant publication bias in this study. The funnel plots (Fig. 8 and Fig. 9) showed the association between the log odds ratio for CHD risk related to ApoB Xbal gene for A/a allele and the standard error of the OR. The shapes of the two funnel plots were roughly symmetrical.

**Table 4:** Results exhibition of Egger’s test in three genetic models

Genetic models	Std	t	p	95%CI
a vs A	1.878	0.72	0.49	−2.84-5.53
aa vs AA/Aa	1.534	0.32	0.76	−3.05-4.02
aa/Aa vs AA	1.728	0.86	0.41	−2.37-5.33

### Sensitivity analysis

**Fig. 10:**
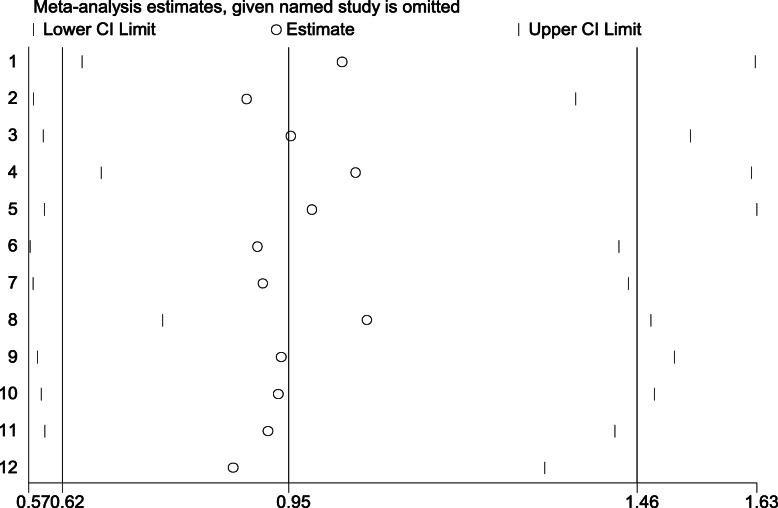
The analysis of sensitivity (impact map of each eligible study, estimated OR and 95% CI assessed by excluding the document)

The OR value obtained by subtracting one document at a time was compared with the combined OR value to analyze the sensitivity of this study. The combined OR estimates after deleting each study could be seen in Table 5 and Fig. 10,and the leave one out OR estimates ranged from 0.87 (1.57–1,32) to 1.06 (0.76–1.48) in allelic comparison(a vs A), respectively,indicating that none of these studies significantly affected the combined OR. One could see that the combined OR estimates after deleting each study were located within 95% CI of the total effect, which demonstrate that none of the studies influence the pooled OR to any significant extent. When the same approach was applied to the other two models, the results were consistent. All the results indicated that the pooled estimates of allelic and other genetic models risks obtained in this study were statistically robust.

**Table 5:** The analysis of sensitivity

Study omitted	Estimate	95% Conf. Interval	
Abd El-Aziz TA	1.03	0.65	1.63
LI.S.H	0.89	0.58	1.37
Huang G	0.95	0.59	1.5
MA.J	1.05	0.67	1.6
Anghebem-Oliveira	0.98	0.59	1.63
BELGIN S. DUMAN	0.90	0.57	1.43
Marcos O. Machado	0.91	0.57	1.45
Luis A. Salazar	1.06	0.76	1.48
VADIM A	0.94	0.58	1.51
Seung Ho Hong	0.93	0.59	1.48
Ju-Pin Pan	0.92	0.59	1.43
YE.P	0.87	0.57	1.32
Combined	0.95	0.62	1.46

Note: to assesse the estimated OR and 95% CI by excluding the document

## Discussion

The ApoB Xbal restriction site gene polymorphism mentioned in this passage is located in the 26th exon of ApoB [26], which turned the code ACC encoding 2488 threonine to ACT,although the amino acid sequence is not changed. However, the X+ allele is produced, which is a rare gene. In the study by YE [12], the X+ allele frequency was significantly higher in the CAD group than in the normal control group. It is speculated that the significance may be a genetic marker that is in linkage with the functional mutation of the ApoB gene itself, leading directly to the formation of atherosclerosis, or by affecting cholesterol. The rate of conversion of esters in HDL and LDL leads to a decrease in high-density lipoprotein levels, thereby promoting the development of atherosclerosis. Using XbaI enzyme to digest the 26th exon of the ApoB gene,and the homozygote X ^+^ X ^+^ with XbaI cleavage site had a lower metabolic rate of LDL than the homozygous X^−^X^−^ without XbaI cleavage site. Further analysis revealed that the A^↓^G mutation occurred in the 13141th base, and the encoded asparagine was replaced by serine. It was found in vitro that the affinity of the apoB protein synthesized by its apoB protein decreased with its corresponding LDL receptor, which affected the metabolic clearance of LDL. Studies have confirmed that LDL-C is an independent risk factor for coronary heart disease. ApoB XbaI gene polymorphism ultimately leads to coronary atherosclerosis by affecting the metabolic clearance of LDL and increasing plasma ApoB levels. This meta-analysis included 12 articles. The specific data included 1596 cases in the coronary heart disease group and 1431 cases in the control group. The results showed that there was no significant correlation between ApoB XbaI gene polymorphism and coronary heart disease.

Coronary heart disease is a multi-gene and multi-factorial disease. Many studies have shown that plasma apolipoprotein B concentration and its gene polymorphism are inextricably linked to the development of coronary heart disease. However, because the observed population has different genetic backgrounds and environmental factors, there are often inconsistencies in the analysis of the association between disease and specific genes, especially for rare alleles with low frequency of distribution. The analysis of observations is subject to sample size. Abd El-Aziz et al. [15] concluded that the ApoB X + allele increased the risk of CHD by affecting the levels of TC and LDL-c by studying the polymorphisms of various apolipoprotein B genes in the Egyptian population; Ma Juan et al. [16] conducted a case-control study of Chinese people suggesting that ApoB Xbal gene polymorphism is a genetic risk factor for CHD, and that allele X+ is an important genetic marker for CHD. Li Shunhui [17] and Ye Ping et al. [12] did similar studies. However, in the study of the Turkish population by Belgin S. Duman [18] and the study by the Ju-Pin Pan [19] on the Chinese population, there was no significant difference in genotype frequencies between the EcoRI and XbaI loci of the Apo B gene between the patient and the control group. Marcos O. Machado et al. [20] used haplotype analysis to study the high risk of Apo B gene and coronary heart disease on the Brazilian population. The results showed that single haplotype X + Del in apo B gene has an effect on lipid metabolism and may contribute to the susceptibility to development of CHD among Brazilian men. Similarly, there are another two studies on the Brazilian population included studies by Luis A. Salazar [21] and Anghebem-Oliveira, MI, [22] but the conclusions were inconsistent. The former showed that the Apo B gene polymorphism was associated with Brazilian Caucasian CAD, and the latter had nothing to do with it. In another study [23] on Russians,it was argued that ApoB genetic polymorphism affects the development of coronary heart disease by affecting the level of internal lipids.

Meta-analysis conducted a secondary analysis of descriptive data has a scientific and rational aspect, but it is also affected by a variety of factors including the amount of selected literature, the size of the total sample size, publication bias, and flaws in the analytical method itself. Through the foregoing description, this study has been proven to be heterogeneous through various methods of heterogeneity test(*p* < 0.05). In the three gene models studied in this paper we tested the heterogeneity, and they all showed significant heterogeneity. Therefore, we used random effect models to combine data, and we performed subgroup analysis according to ethnicity to explore the sources of heterogeneity. Interestingly, in the recessive genetic models, the heterogeneity between Asians and Caucasians was very different, seemingly indicating that this subgroup was a source of heterogeneity. However, we could also see that in the Asian subgroup,two piece of literature [12, 19] were excluded from analysis because they had no recessive genotypes, which may cause false positive, so the lack of clarity needs to be explored further. Although no obvious sources of heterogeneity have been found in other genetic models,we used random effect models, related sensitivity analysis and subgroup analysis to control and explain. No obvious source of heterogeneity was found, but our conclusions were still reliable from a methodological perspective.Some people think that the existence of this heterogeneity is due to the time of publication of the literature, the grouping of the research, the characteristics of the research object, and so on. However, due to the limited literature used for this study, the small sample size and the differences between ethnic groups may affect the accuracy of the results.

With regard to this meta-analysis, we should recognize the existence of certain restrictions. First of all, this article only includes twelve articles that we can read in full, and thus misses some eligible articles that have not been published or have been published but where we cannot read the full text. Secondly, this paper mainly studies the influence of single gene loci and ignores the result of the interaction of multiple genes. Thirdly, we can only get the data from each study by reading the full text, but we cannot get the original research data and research methods and thus cannot make a comprehensive comparison. In addition, we only perform subgroup analysis in terms of ethnicity, without considering other subgroups,such as sample size, age, gender, etc. This caused us to have no clear source of heterogeneity. Finally, the sample sizes in some studies are small in some studies, which may reduce the statistical power.

## Conclusion

In conclusion, we cannot see any significant correlation between the ApoB XbaI gene polymorphism and the occurrence and development of coronary heart disease from the statistical data. When we performed subgroup analysis, we found that Caucasians carrying the aa genotype were less susceptible to CHD in recessive genetic models. However, subgroup analysis reduces sample sizes which means errors may increase.There was insufficient data to fully confirm the association. In our clinical life, dyslipidemia is an important risk factor for atherosclerotic coronary heart disease(ASCVD). The significant positive correlation between plasma apolipoprotein B and LDL-C indicates that it is often an important predictor of CHD, and the risk-related relevance between its polymorphisms and coronary heart disease requires more large-scale original studies to be further validated.

## Data Availability

Data sharing not applicable to this article as no datasets were generated or analysed during the current study.

## Abbreviations

CHD: Coronary heart disease
CAD: Coronary atherosclerotic disease
Apo B: Apolipoprotein B
HWE: Hardy-Weinberg equilibrium
LDL: Low-density lipoprotein
